# Assessment of Murine Colon Inflammation Using Intraluminal Fluorescence Lifetime Imaging

**DOI:** 10.3390/molecules27041317

**Published:** 2022-02-15

**Authors:** Alba Alfonso-Garcia, Stephanie A. Cevallos, Jee-Yon Lee, Cai Li, Julien Bec, Andreas J. Bäumler, Laura Marcu

**Affiliations:** 1 Biomedical Engineering Department, University of California, Davis, CA 95616, USA; caili@ucdavis.edu (C.L.); jbec@ucdavis.edu (J.B.); lmarcu@ucdavis.edu (L.M.); 2 Medical Microbiology and Immunology Department, University of California, Davis, CA 95616, USA; vanialex2@gmail.com (S.A.C.); jeylee@ucdavis.edu (J.-Y.L.); ajbaumler@ucdavis.edu (A.J.B.)

**Keywords:** fluorescence lifetime imaging, autofluorescence, inflammatory bowel disease, colon dysbiosis

## Abstract

Inflammatory bowel disease (IBD) is typically diagnosed by exclusion years after its onset. Current diagnostic methods are indirect, destructive, or target overt disease. Screening strategies that can detect low-grade inflammation in the colon would improve patient prognosis and alleviate associated healthcare costs. Here, we test the feasibility of fluorescence lifetime imaging (FLIm) to detect inflammation from thick tissue in a non-destructive and label-free approach based on tissue autofluorescence. A pulse sampling FLIm instrument with 355 nm excitation was coupled to a rotating side-viewing endoscopic probe for high speed (10 mm/s) intraluminal imaging of the entire mucosal surface (50–80 mm) of freshly excised mice colons. Current results demonstrate that tissue autofluorescence lifetime was sensitive to the colon anatomy and the colonocyte layer. Moreover, mice under DSS-induced colitis and 5-ASA treatments showed changes in lifetime values that were qualitatively related to inflammatory markers consistent with alterations in epithelial bioenergetics (switch between β-oxidation and aerobic glycolysis) and physical structure (colon length). This study demonstrates the ability of intraluminal FLIm to image mucosal lifetime changes in response to inflammatory treatments and supports the development of FLIm as an in vivo imaging technique for monitoring the onset, progression, and treatment of inflammatory diseases.

## 1. Introduction

Inflammatory bowel disease (IBD) is expected to affect 10 million people in the Western world by 2030 [1]. IBD progressively develops over years, carrying a big burden on quality of life and healthcare costs [2,3]. Unfortunately, IBD is challenging to diagnose as it presents with variable clinical symptoms that are often vague and overlapping with those of other gastrointestinal diseases [4,5]. Despite the fact that treatments for inflammatory disease have improved in the last decades, screening remains the strategy with the best real impact on reducing the incidence and early detection is key in preventing complications (i.e., IBD-induced colorectal cancer) [6,7].

Current diagnostic tools perform best at detecting advanced stages of IBD with overt inflammation. The most established methods to diagnose IBD are either non-invasive and indirect (i.e., stool calprotecting or serological biomarkers) [4], or destructive (biopsy-based). Indirect methods have rather low specificity, and biopsies mainly detect significant inflammation based on histopathological findings. Endoscopic imaging has gained traction as an in situ method to visualize inflammation [8,9,10] and is instrumental to diagnosing IBD and its subtypes (ulcerative colitis and Crohn’s disease) [11,12]. Fluorescence molecular endoscopy that uses fluorescent probes targeting cancerous tissue (e.g., bevacizumab-800CW and EMI-137) has shown improved rates of polyp detection over white light endoscopy alone [13,14]. However, most imaging approaches lack sensitivity to early inflammatory events and require exogenous contrast agents [10,15]. To date, most diagnosis are made by exclusion of other disease and involve a combination of tests [16], which imply delays in the final results. Low-grade inflammation remains elusive to diagnose. New endoscopic strategies to directly detect early signs of inflammation on the colon mucosa would improve patient’s prognosis and alleviate the health care associated costs of disease management.

Early manifestations of inflammation include colon dysbiosis. Dysbiosis represents an imbalance in the gut microbiota [17,18] and is associated with a dysfunctional colon epithelium [19]. Under dysbiosis, regular epithelial function based on high mitochondrial oxygen consumption shifts to aerobic glycolysis. Such metabolic shift leads to an increased leakage of oxygen into the colon lumen, which maintains the dysbiotic state and exacerbates chronic inflammation and health-compromising disease. The early shift in epithelial metabolism, however, offers an opportunity to design early detection methods through fluorescence lifetime imaging (FLIm) of tissue autofluorescence.

At the cellular level, a metabolic shift from oxidative phosphorylation to glycolysis results in a decrease in the fluorescence lifetime as the ratio of free to bound NADH (nicotinamide adenine dinucleotide) increases [20,21]. A decrease in cellular fluorescence lifetime was observed in polarized macrophages [22,23] that undergo a similar metabolic switch under inflammation as seen in epithelial colonocytes [19]. Differentiating colonocytes also exhibit an increase in lifetime as they move from undifferentiated to terminally differentiated cells and their metabolic activity changes from glycolysis-based to fatty acid oxidation [21,24]. In thick tissues, the measured fluorescence will also have contribution from structural proteins in the submucosa (i.e., collagen and elastin), even under near-UV excitation with a shallow penetration depth, and other tissue components (i.e., porphyrins) [25,26]. Therefore, the sensitivity of this approach to metabolic changes remains to be evaluated.

Fluorescence lifetime is sensitive to the tissue microenvironment, and thus it adds a new dimension to characterize malignant tissue. Compared to intensity measurements, lifetime is a more robust metric because it is less sensitive to artifacts created by imaging parameters such as variations in probe-to-tissue distance. Additionally, the use of tissue autofluorescence as a source of contrast, as opposed to exogenous fluorescent probes, simplifies preparation protocols and mitigates potential cytotoxic effects. Autofluorescence lifetime imaging has already been used on thick tissue to detect advanced colon lesions [12,27,28,29], where early work focused on identifying different polyp types in patients with colon cancer [30]. Most FLIm studies were typically performed ex vivo through wide-field imaging approaches [12,27,28,29,31], or in vivo as point-based spectroscopic measurements [27].

The objective of this study was to test the feasibility of label-free intraluminal FLIm to detect the inflammatory response of thick tissue. This study was conducted on freshly excised mice colons with severe inflammation as a result of DSS-induced colitis. The imaging approach relied on helical scanning (pullback and rotation) with a side-viewing optical fiber terminated by a freeform focusing micro-optic [32], and optical contrast was based on time-resolved tissue autofluorescence that do not require exogenous contrast agents. As a result, the full mouse colon (50 to 80 mm) was scanned in less than 10 s in an in vivo-friendly imaging mode. This FLIm instrument design enables minimally invasive macro-endoscopic imaging that could become a tool to study and monitor disease onset, progression, and treatment.

## 2. Results

### 2.1. Autofluorescence Properties Differ along Distinct Anatomical Regions within The Colon

**Figure 1:**
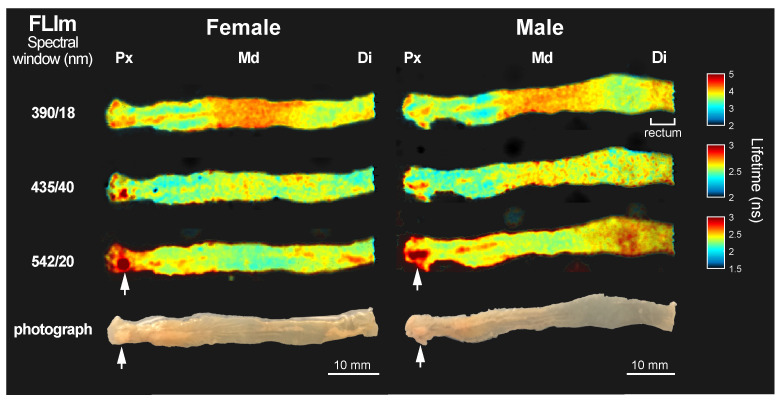
Fluorescence lifetime maps of exposed colon lumen from two representative samples, a female and a male subject, at spectral windows 390/18 nm, 435/40 nm, and 542/20 nm. The corresponding photograph of each colon luminal surface is included in the bottom row. The color bar for the lifetime maps of each spectral band has been adjusted to maximize contrast. White arrowheads point to fat accumulations in the samples. Three anatomical regions are identified as proximal (Px), middle (Md), and distal (Di) colon. This male sample also shows distinct lifetime in the rectum.

Colons of untreated mice were imaged to obtain a reference fluorescence lifetime baseline of the luminal surface. Figure 1 shows representative fluorescence lifetime maps from a female and a male specimen. The colons were oriented from the proximal side, cut under the cecum, to the distal end, which contains the rectum. The results were comparable among all 6 tested mice (*n* = 3 female, and *n* = 3 male).

The average lifetime changed along the length of the colons in a spectrally dependent manner. At 390 nm, the longest average lifetime was in the middle (Md) region of the colon ($\tau_{Md}=4.11\pm 0.08$ ns). The rectum showed very similar values. The proximal (Px) and distal (Di) colon had shorter mean average lifetime values than the middle region, but comparable to one another ($\tau_{Px}=3.60\pm 0.20$ ns and $\tau_{Di}=3.74\pm 0.11$ ns). At 435 nm, the middle and distal colon had comparable mean average lifetimes ($\tau_{Md}=2.55\pm 0.09$ ns and $\tau_{Di}=2.55\pm 0.08$ ns), whereas the proximal colon exhibited slightly faster decays ($\tau_{Px}=2.46\pm 0.12$ ns). At 542 nm, the trends were reversed from the 390 nm images. The longest mean average lifetime values were on the proximal ($\tau_{Px}=2.39\pm 0.18$ ns) and distal ($\tau_{Di}=2.44\pm 0.08$ ns) colon, and the middle colon had overall shorter average lifetime ($\tau_{Md}=2.28\pm 0.06$ ns). The longest lifetime values (τ∼4 ns, white arrowheads in Figure 1) appeared sporadically in the images at the highest wavelength range, which were consistent with accumulations of fat. These accumulations of fat were excluded in the mean average lifetime calculations from the different regions.

### 2.2. The Epithelial Layer Influenced Tissue Autofluorescence Properties

**Figure 2:**
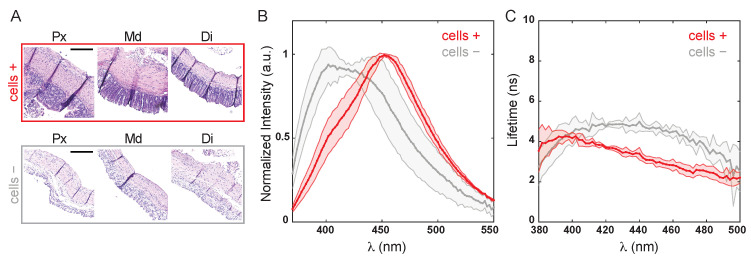
Fluorescence tissue properties of mice colon before (cells +) and after (cells −) removing endothelial colonocytes. (**A**). Hematoxylin and Eosin (H&E) stain of tissue sections from the proximal (Px), middle (Md), and distal (Di) colon. Scale bar = 200 μm. (**B**). Normalized autofluorescence emission spectra and (**C**) lifetime spectra.

The thickness of the epithelial layer composed of colonocytes ranged from ∼100 to 400 μm depending on the animal and anatomical area (e.g., Figure 2A: Px∼220 μm, Di∼130 μm). To verify that FLIm with 355 nm excitation, which has a shallow penetration depth, was sensitive to the epithelial layer, spectral and lifetime properties were measured before and after removing colonocytes from the tissue (Figure 2). Figure 2A shows H&E stain images of representative proximal, middle, and distal colon, before (cells +) and after (cells −) removing the colonocytes.

Figure 2B,C shows the normalized average colon spectra and the lifetime lifetime spectra before and after the removal of the epithelial layer. The intensity spectrum of intact tissue peaked at 454 ± 5 nm (cells +), and the emission maximum shifted to 417 ± 13 nm after removing the cells (cells −). These broad spectra are characteristic of tissue autofluorescence, with a prominent contribution of cellular autofluorescence from metabolic co-factors (i.e., NADH with emission maximum around 460 nm). A shoulder around 400 nm in the intact spectrum is likely caused by fluorescence of structural proteins components such as collagen and its crosslinks, and elastin. This shoulder becomes a prominent peak once the cells are removed and the submucosal tissue is exposed. The fluorescence lifetime (Figure 2B) increased in the 400 to 500 nm spectral region from 2–3 ns (cells +) to over 4 ns after cell removal (cells −). Taken together, these results confirmed that the fluorescence originated in the cellular epithelial layer greatly contributed to the detected tissue autofluorescence. However, additional contribution from extracellular structural proteins in the submucosa to the overall tissue autofluoresnce cannot be discarded, especially in the shorter spectral band under 400 nm.

### 2.3. Colon Fluorescence Lifetime Was Sensitive to Tissue Inflammation

**Figure 3:**
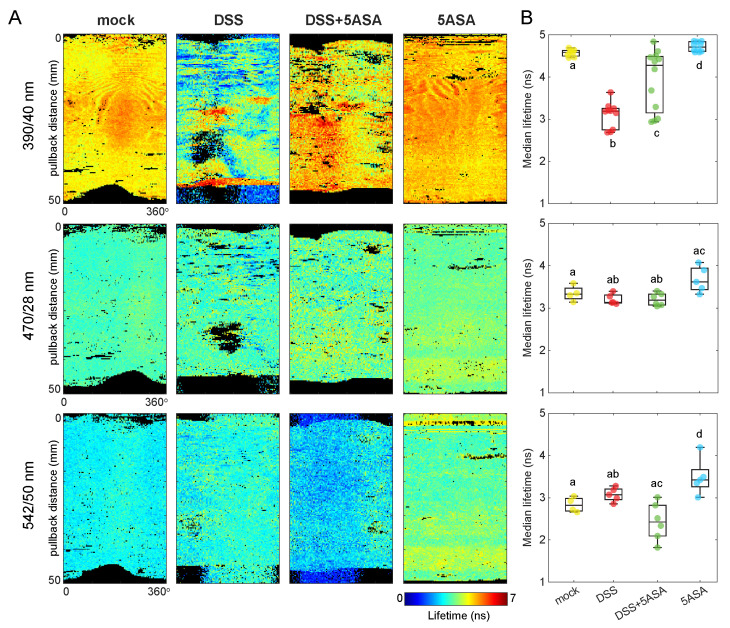
(**A**) Representative fluorescence lifetime maps for each treatment group in every spectral band used for this study. Images were acquired intraluminally and are shown here as flat 2D maps of the luminal surface from the proximal (top) to the distal (bottom) ends of the colon for ease of interpretation. Black pixels indicate outliers (lifetime below 0.25 percentile or above 0.75 percentile). (**B**) Median average fluorescence lifetime from each imaged colon for each condition and spectral band. Pairwise Mann–Whitney U test was applied to test statistical differences between groups.

To test the ability of FLIm to detect inflammation we used dextran sodium sulfate (DSS) to induce colitis on the mouse colon and 5-aminosalicylic acid (5-ASA), a PPAR-γ (peroxisome proliferator-activated receptor gamma) agonist that acts on the colon epithelial cells, to mitigate the inflammatory effects. Figure 3A shows representative average lifetime maps in each spectral band for mice treated with DSS, DSS + 5ASA, and the corresponding controls (mock and 5ASA). The median of the average lifetime values in each image was computed and used as the unit of comparison in Figure 3B.

The highest contrast in average fluorescence lifetime between treatment groups was observed at 390 nm. The fluorescence lifetime of mock-treated colons agreed with the baseline results (Figure 1). The median and interquartile range of all mock-treated colons at 390 nm was $\tau_{\mathrm{mock}}^{390\mathrm{nm}}=4.59\left[4.51,4.63\right]$ ns. The DSS-treated group had the shortest overall fluorescence decays at this wavelength ($\tau_{\mathrm{DSS}}^{390\mathrm{nm}}=3.19\left[2.85,3.25\right]$ ns). Mice treated with DSS + 5ASA showed a wide range of lifetime responses ($\tau_{\mathrm{DSS}+5\mathrm{ASA}}^{390\mathrm{nm}}=4.28\left[3.22,4.48\right]$ ns), in between the DSS- and the mock-treated groups. Such a variation may depend on the efficacy of treatments on an individual basis. Finally, the control group with only 5ASA exhibited very similar responses animal to animal, but it had a significantly longer lifetime response ($\tau_{5\mathrm{ASA}}^{390\mathrm{nm}}=4.71\left[4.62,4.82\right]$ ns) compared to the other groups, including mock-treated mice.

At longer wavelengths, which capture cellular contributions, the differences in overall lifetime response were mild. At 470 nm, the DSS and DSS + 5ASA groups showed comparable lifetime responses ($\tau_{\mathrm{DSS}}^{470\mathrm{nm}}=3.14\left[3.13,3.28\right]$ ns and $\tau_{\mathrm{DSS}+5\mathrm{ASA}}^{470\mathrm{nm}}=3.19\left[3.08,3.32\right]$ ns), that were shorter than the control groups ($\tau_{\mathrm{mock}}^{470\mathrm{nm}}=3.33\left[3.26,3.41\right]$ ns, and $\tau_{5\mathrm{ASA}}^{470\mathrm{nm}}=3.62\left[3.47,3.90\right]$ ns). At 542 nm, DSS-treated colons ($\tau_{\mathrm{DSS}}^{542\mathrm{nm}}=3.07\left[2.99,3.19\right]$ ns) showed longer lifetimes than mock-treated ($\tau_{\mathrm{mock}}^{542\mathrm{nm}}=2.82\left[2.70,2.95\right]$ ns), although the differences were non-significant. The DSS + 5ASA group, however, was statistically different from the DSS group, with shorter lifetime values closer to mock levels ($\tau_{\mathrm{DSS}+5\mathrm{ASA}}^{542\mathrm{nm}}=2.43\left[2.16,2.75\right]$ ns). In this spectral band, the 5ASA control group was significantly different than all the rest, with the longest lifetime response ($\tau_{5\mathrm{ASA}}^{542\mathrm{nm}}=3.42\left[3.35,3.49\right]$ ns).

**Figure 4:**
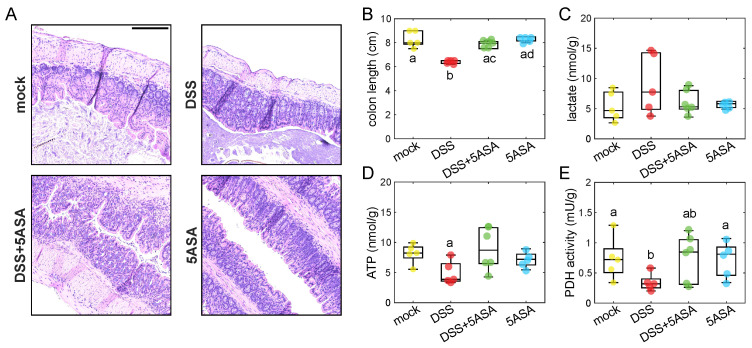
(**A**) Representative H&E images of distal colon sections from each group. Scale bar = 200 μm. Biochemical measurements on the imaged colons include colon length (**B**), intracellular lactate (**C**), ATP levels (**D**), and PDH activity (**E**). Pairwise Mann–Whitney U test was applied to test statistical differences between groups.

Physical and biochemical markers were also measured in the harvested colons (Figure 4). DSS-induced colitis resulted in epithelial hyperplasia (Figure 4A). Moreover, DSS-treated colons were shorter than the rest (Figure 4B), which is a marker of inflammation. Metabolic indicators measured on isolated colonocytes indicated a switch to aerobic glycolysis on the DSS-treated colons, characterized by a non-significant increase of intracellular lactate levels (Figure 4E) and a significant decrease of ATP levels (Figure 4C) and PDH activity (Figure 4D). Colonocytes from mice treated with DSS + 5ASA showed values closer to the control groups, indicating a maintenance of the β-oxidation phenotype.

## 3. Discussion

The imaging approach presented in this study employed time-resolved pulse sampling FLIm for fast imaging of large and tick tissue samples. The mucosal surface of mouse colons, 50 to 80 mm in length, were imaged in less than 10 s. To achieve helical intraluminal imaging, the FLIm system was mounted on a pullback stage coupled to a side-viewing endoscopic probe through a rotary collimator. This tool enabled non-destructive macroscopic imaging of the colon mucosal surface, that unlike homogenized biochemical assays, provides a spatially-resolved method to map the effects of inflammation in situ.

The current results demonstrate that FLIm with 355 nm excitation was sensitive to the fluorescence from the epithelial layer and show that autofluorescence lifetime varies along the distinct anatomical regions of the colon. Additionally, these results demonstrate that FLIm is sensitive to severe inflammation, as lifetime values under DSS-induced colitis were significantly altered compared to reference mock levels, and comparable to mock under 5-ASA treatment. The changes in fluorescence lifetime qualitatively aligned with changes in inflammatory markers including colon length and intracellular metabolic biomarkers (lactacte, ATP, and PDH activity) that confirmed the efficacy of treatments. These results encourage further correlation studies between the tissue inflammatory response and its autofluorescence properties (spectra and lifetime).

As part of the study, we established a reference lifetime baseline from the distinct average lifetime along the length of healthy colons (Figure 1) associated with three well-known anatomical regions, namely proximal, middle, and distal colon. The different lifetime values may result from the distinct composition and arrangement of cells in the mucosa and structural proteins in the submucosal layer along the colon length [33] that directly impact the fluorophores environment. The three regions present a distinct response to inflammatory processes (i.e., DSS-induced colitis largely affects the distal colon [34]); therefore, a properly characterized spatially-resolved baseline is necessary to quantify tissue lifetime changes in each region independently, which will lead to more accurate diagnosis. Results were consistent across six measured animals (three males and three females) suggesting robust and reproducible measurements can be expected from tissue autofluorescence lifetime of healthy tissue.

Sensitivity to the epithelial layer was verified by time-resolved spectroscopy performed on the tissue before and after removing colonocytes, which directly exposed the underlying submucosa composed of structural proteins of the extracellular matrix. The cellular contribution was the strongest at 470/28 nm and 542/50 nm, as the NADH and flavin emissions peak at 460 nm and 525 nm, respectively [35]. Therefore, their removal resulted in a spectral blue shift (Figure 2). However, contribution to the overall fluorescence at 390/40 nm from crosslinks found in structural proteins (i.e., pyridinoline) is expected, and bleed-through into the 470/28 nm channel cannot be discarded, which will impact the interpretation of the results. The increase in lifetime after removing the epithelial layer further confirmed the contribution of fluorescence from colonocytes to the measured lifetime in intact tissue. The contribution from cellular fluorescence was more noticeable starting at ∼400 nm, with lifetime values around 2 to 3 ns in intact tissue, whereas exposed submucosa exhibited longer-lasting decays (∼5 ns) characteristic of the structural proteins [36].

The DSS-induced colitis model of severe inflammation [37] was employed to evaluate the feasibility of intraluminal FLIm to detect colon inflammatory disease. A significant decrease in fluorescence lifetime was consistently observed at 390/40 nm (Figure 3), which could be explained by the severe physical alterations of the tissue structure under DSS treatment (Figure 4A,B). DSS-induced colitis was also expected to reduce mitochondrial activity in the colonocytes [38], forcing a metabolic switch from β-oxidation to aerobic glycolysis. Albeit non-significant, the shorter lifetime values observed in the DSS group compared to the control at 470/28 nm (Figure 3) agreed with the higher levels of intracellular lactate and lower levels of ATP and PDH activity, which indicate the expected metabolic shift towards a glycolysis (Figure 4C–E). This faster decays could be explained by an increase of free NADH with respect to protein-bound NADH. Free NADH, with a characteristic short lifetime, is abundant in glycolytic phenotypes that bypass the electron transport chain [20]. Additional metabolic information relevant to the inflammatory state of the tissue could be obtained from the redox state of the epithelium (i.e., NAD+/NADH), by incorporating calibrated intensity-based measurements to quantify the optical redox ratio from NADH and FAD intensities [20].

The effects of DSS were mitigated by 5-aminosalicylic acid (5-ASA), a PPAR-γ (peroxisome proliferator-activated receptor gamma) agonist that acts on the colon epithelial cells. 5-ASA is known to restore mitochondrial bioenergetics and provides an overall defense against DSS-induced colitis [39]. The autofluorescence lifetime from colons treated with DSS and 5-ASA was closer to mock levels than to DSS-treatment alone, indicating the treatments were effective. Treatment efficacy was further corroborated by the return to β-oxidation characterized by lower lactate levels (Figure 4C), and higher ATP and PDH activity levels (Figure 4D–E), as well as longer colon lengths (Figure 4B) compared to the DSS group.

5-ASA and its metabolite N-acetyl-5-ASA (Ac-5-ASA) emit a weak but broad fluorescence that peak at ∼500 nm and ∼440 nm, respectively, when excited with UV light [40]. This fluorescence was likely responsible for the increase in fluorescence lifetime observed in the 5-ASA control group. It is unclear how the additional fluorescence of 5-ASA affected the lifetime of DSS-5ASA treated colons (Figure 3). DSS-induced colitis decreases 5-ASA metabolism in mice [41], therefore reduced contribution of Ac-5-ASA fluorescence was expected in this group. Additionally, the changes of fluorescence emission and lifetime properties of 5-ASA as it acts on inflamed colons (i.e., due to quenching and other conformational changes when it binds to enzymes) are unknown. Further studies will need to consider this contribution with more detail to understand potential confounding effects between the fluorescence of the molecule itself and epithelial fluorescence related to bioenergetics.

FLIm is sensitive to changes in the epithelial metabolism that are connected to alterations in the microbiota, which play a key role in IBD development. Dysbiotic colons (i.e., with IBD, or in the model used here colons treated with DSS) exhibit an increased population of *E. coli* Nissle wild type because of the available oxygen leaked from the dysfunctional epithelium, which they can use as an electron acceptor. This expansion of E.coli wild type has been demonstrated in the DSS model with an increased competitive index between wild type and cydA mutant, which cannot respire oxygen [39]. Additional 5-ASA treatment reduced the competitive index, as the restoration of epithelial function limited the availability of oxygen in the lumen. Quantitative measurements of epithelium oxygenation with phosphorescence lifetime imaging (PLIM) with oxygen-sensitive probes have also shown both the reduced oxygen consumption rate in colon epithelium and increased lumen oxygenation in DSS-treated mice [42]. These reported findings together with our current results suggest that FLIm can be a useful additional tool to achieve a mechanistic understanding of dysbiosis in IBD in the future.

In summary, the current results demonstrate the use of intraluminal FLIm for detecting the tissue response to inflammation and treatment. The detected fluorescence lifetime combines signals from epithelial energy metabolism and structural components of the extracellular matrix in the submucosa. Questions to address in future studies include the specific weight of each component to the fluorescence response, and the sensitivity of FLIm to low-grade inflammation. These results obtained with intraluminal FLIm ex vivo measurements encourage future studies in vivo, which is the most relevant scenario for metabolic imaging. This instrumental design enables rapid screening and longitudinal monitoring of disease onset, progression, and treatment, thereby addressing some of the current needs for non-invasive imaging [43].

## 4. Materials and Methods

### 4.1. Animal Models

**Figure 5:**
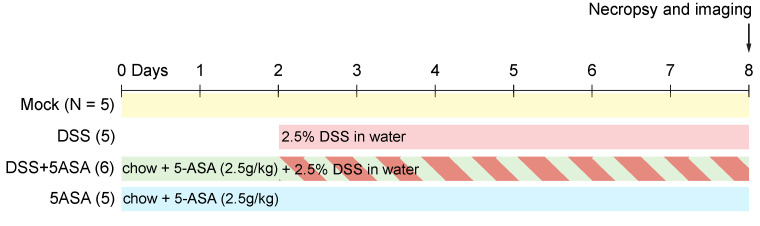
Experimental design. Mice were divided into four treatment groups: mock-treated (N = 5), DSS (N = 5) mice received 2.5% DSS in water for 6 days starting at the indicated time, DSS + 5-ASA (N = 6) mice received 2.5g/kg 5-ASA in chow for 8 days and 2.5% DSS in water for 6 days starting at the indicated time, and 5-ASA (N = 5) mice received just the chow with 2.5g/kg of 5-ASA during the duration of treatment.

All experiments in this study were approved by the Institutional Animal Care and Use Committee at the University of California at Davis. C57BL/6J wild type mice, aged 8 to 10 weeks, were obtained from The Jackson Laboratory. Male (*n* = 3) and female (*n* = 3) mice were used for the preliminary evaluation of colon fluorescence and no differences were observed. The experiment on colon inflammation was done with male mice (*n* = 4–6 per group). Mice were acclimated for a week before starting any treatments and humanely euthanized at the end of each treatment. Imaging was done immediately after necropsy (Figure 5).

### 4.2. DSS Treatment

Mice were treated with dextran sodium sulfate (DSS) administered ad libitum as 2.5% in drinking water for 6 days, when colons were harvested upon necropsy.

### 4.3. 5-ASA Treatment

Mice were treated with 5-aminosalicylic acid (5-ASA) administered as 2.5 g/kg in the chow. A control group was kept on the 5-ASA supplemented diet for 9 days, and the treatment group was administered 2.5% DSS starting two days after initiating the chow. Colons were harvested upon necropsy, 6 days post DSS treatment.

### 4.4. Colonocyte Isolation

The colon and part of cecum were opened lengthwise and cut into 2 to 4 cm pieces, collected in 15 mL of ice-cold 1× RPMI 1640 buffer (Gibco, Waltham, MA, USA) in a 50 mL Falcon tube and cleaned with 20 mL of ice-cold 1× Dulbecco’s phosphate-buffered saline (DPBS; Gibco) in another 50 mL Falcon tube. The tissue was then placed into 15 mL conical centrifuge tubes filled with 10 mL of ice-cold dissociation reagent 1 (30 mM EDTA, 1.5 mM dithiothreitol (DTT), diluted into 1× DPBS) and placed on ice for 20 min. Tissues were then placed into a 15 mL conical centrifuge tube filled with 6 mL of warm (37 °C) dissociation reagent 2 (30 mM EDTA, diluted into 1× DPBS) and incubated for 10 min at 37 °C. After this incubation, tubes were shaken vigorously for 30 s to detach the epithelium from basement membrane, for a total of about 80 to 90 shake cycles. Remnant intestinal tissue was removed, and pellet cell solution was centrifuged at 800× *g* for 5 min at 4 °C. Supernatant was removed, and the cell pellet was re-suspended in radioimmunoprecipitation assay (RIPA) buffer for metabolism analysis.

### 4.5. Lactate Measurements

For measuring intracellular lactate levels, primary colonocytes were isolated as described above and deproteinized by using a Deproteinizing Sample Preparation Kit (Biovision, Milpitas, CA, USA). Lactate measurements in colonocyte lysates were performed by using the Lactate Colorimetry Assay Kit 2 (Biovision, Milpitas, CA, USA) following the manufacturer’s instructions.

### 4.6. ATP Measurements

For measuring intracellular ATP levels, primary colonocytes were isolated as described above and deproteinized by using a Deproteinizing Sample Preparation Kit (Biovision, Milpitas, CA, USA). ATP measurements in colonocyte lysates were performed by using ATP Colorimetry Assay Kit (Biovision, Milpitas, CA, USA) according to the manufacturer’s instructions.

### 4.7. Pdh Activity Measurements

For measuring PDH activity, primary colonocytes were isolated as described above and lysed with RIPA buffer, and cellular debris were removed by centrifugation for 5 min at 13,000 rpm at room temperature. The supernatant was used to measure PDH activity using the PDH Activity Assay Kit (Biovision, Milpitas, CA, USA) according to the manufacturer’s instructions.

### 4.8. Histology Images

The proximal and distal ends of the colon were fixed in 10% phosphate-buffered formalin, and 5 μm sections of the tissue samples were stained with Hematoxylin and Eosin (H&E) following routine procedures. Images were taken using a Keyence BZ-X710 microscope (Keyence, Itasca, IL, USA) with a Plan Apo λ 10×/0.45NA objective lens (Nikon Instruments, Melville, NY, USA).

### 4.9. Time-Resolved Fluorescence Spectrometer

Tissue autofluorescence was excited with 355 nm pulsed laser (<0.6 ns per pulse, >2 μJ per pulse, repetition rate 4 kHz; TEEM photonics STV-02E, Meylan, France). A 400 μm core diameter multimode fiber placed perpendicular to the sample was used to deliver and collect the light. A second multimode fiber (600 μm in core diameter) was used to guide the tissue autofluorescence to a monochomator (MicroHR, Horiba, Kyoto, Japan). Light was detected with a gated microchannel plate photomultiplier tube (R5916U-50, Hamamatsu, Hamamatsu, Japan) connected to a high-speed amplifier (C5594, Hamamatsu), and digitized by an oscilloscope (DPO7254, Tektronix, Beaverton, OR, USA). The laser power at the sample plane was kept under 5 mW. Fluorescence decays were acquired from 370 to 600 nm with 2 nm increments and a 0.08 ns resolution. Each data point was an average of 64 decays.

Data were acquired from three points along the length of the colon corresponding to the proximal, the middle, and the distal ends. Three measurements were taken on each position. The results presented in Figure 2 represent the mean (solid thick line) over 4 independent samples (2 female and 2 male) and their corresponding standard deviation (shaded area). Samples were kept in phosphate buffer saline (PBS) for the duration of imaging.

### 4.10. Fluorescence Lifetime Imaging (FLIm) Instruments

Two different custom fluorescence lifetime imaging (FLIm) devices were used in this study. Both FLIm devices implemented a pulse sampling approach that enables rapid image acquisition under room-light illumination [44].

Planar images of exposed lumens were acquired with a FLIm apparatus previously described [45] that employs a 355 nm pulsed laser (pulse duration < 0.6 ns, pulse energy > 2 μJ, repetition rate 4 kHz; TEEM photonics STV-02E, Meylan, France). The laser power at the sample plane was kept under 5 mW. Tissue autofluorescence was spectrally resolved into three spectral bands (390/18 nm, 435/40 nm, 542/20 nm). Optical signals from each band were coupled into optical fibers of different length leading to a single microchannel plate photomultiplier tube (MCP PMT, R3809U-50, Hamamatsu, Japan). The signal was further amplified by a transimpedance amplifier (Newport 2001-FS, Irvine, CA, USA) and sampled by a high-speed digitizer (12.5 GS/s, 3 GHz bandwidth; NI PXIe-5185, National Instruments, Austin, TX, USA). The temporal resolution was of 0.08 ns. Fluorescence lifetime maps were acquired by raster scanning a multimode optical fiber perpendicular to the exposed lumen surface with square pixels of 200 μm × 200 μm. An averaging factor of 32 decays was applied to each pixel.

**Figure 6:**
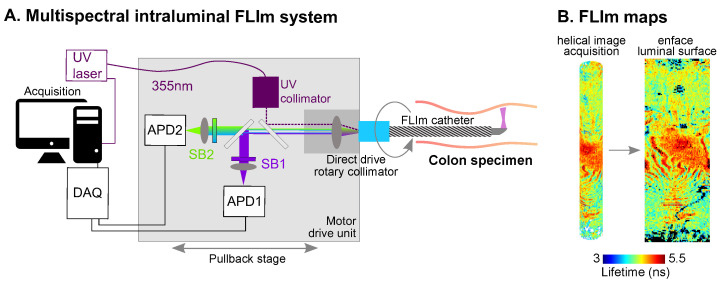
(**A**) Two-channel, APD-based intraluminal FLIm instrument. (**B**) Representative fluorescence lifetime map of a mice colon acquired intraluminally and flattened image as shown in subsequent figures. UV: ultraviolet; APD: avalanche photodiode; SB: spectral band; DAQ: data acquisition board.

Intraluminal images of colons were acquired with a prototype version of an avalanche photodiode (APD)-based multispectral FLIm device [46] (Figure 6A). The excitation source was a 355 nm pulsed laser (repetition rate 20 kHz, pulse duration < 0.08 ns, pulse energy > 400 nJ, Fianium, Southampton, UK). The laser power at the sample plane was kept under 5 mW. Tissue autofluorescence was spectrally resolved into three bands slightly different than the previous setup (390/40 nm, 470/28 nm, 542/50 nm). Intraluminal helical scans of the sample were performed using a custom imaging catheter and motor drive unit. The catheter consisted of an imaging core, composed of a 100 μm core multimode fiber terminated with a freeform reflective micro-optic [32] and enclosed into a double-layer torque coil (Asahi-Intec, Aishi, Japan), and a sheath composed of a 4-Fr braided PEBAX shaft (Merit medical, South Jordan, UT, USA), terminated with an optically transparent polymethylpentene 1.25 mm outer diameter imaging section (Lubrizol, Wickliffe, OH, USA). The motor drive unit was composed of a fiber optic rotary joint and catheter pullback mechanism, excitation laser collimator, spectral separation optics, and APD photodectector modules (modified APD430A2, Thorlabs, Newton, NJ, USA). The signal from the APD was sampled using a 6.25 GS/s digitizer (NI5185, National Instruments, Austin, TX, USA). Pullback speed was ∼10 mm/s. Intraluminal images were reconstructed based on the point measurement rate (20 kHZ) and the catheter rotation speed (2000 rpm), and displayed as en face images (Figure 3A and Figure 6B).

All imaging parameters were chosen such that the energy of the light and exposure duration were well below the ANSI limits for thermal tissue damage (i.e., ANSI Z136.1 [47]).

### 4.11. Spectral Measurements and Imaging of Colon Tissue

Upon necropsy, colon samples were rinsed with phosphate-buffered saline (PBS) to eliminate residual feces. For spectral evaluation and imaging of the exposed lumen, colons were longitudinally cut open. Samples were imaged with the fiber-based FLIm instrument and analyzed with a time-resolved fluorescence spectrometer. Two sets of spectroscopic measurements were acquired, one before and one after removing the epithelial layer from the colon tissue, as described above. For intraluminal imaging, colons were scanned twice with an imaging catheter connected to the 2-channel FLIm instrument to acquire images in three different spectral channels (set 1: 390/40 nm and 470/28 nm; set 2: 390/40 nm and 542/50 nm). We included 2 data points for the 390 nm band in Figure 3B. Samples were kept in PBS during all imaging procedures and placed in ice-cold 1X RPMI 1640 (Gibco) buffer after imaging for subsequent biochemical processing.

### 4.12. Image Analysis and Statistics

The average fluorescence lifetime was computed for each pixel as the expectation value of the probability density function of the fluorescence decay [35]. Fluorescence decays were reconstructed through a constrained least-squares deconvolution with Laguerre expansion as previously described [46,48]. Briefly, for each pixel, the fiber background was first subtracted from the measured signal, and the instrument response function (IRF) was deconvolved from the signal using a constrained least-square deconvolution algorithm with Laguerre expansion to extract the average lifetime. For the evaluation of colon images, pixels with a signal-to-noise ratio below 25 were discarded. The median fluorescence lifetime per colon was computed for a direct comparison between the imaging results and the biochemical analysis. Non-parametric Mann–Whitney U test was used to determine statistically significant differences between the treatment groups of imaging and biochemical metrics. Different letters indicate groups with significantly different metrics (*p* < 0.05). Boxplots (*n* = 4–6) represent the median and the interquartile range of each treatment group for the indicated metric.

## 5. Conclusions

This study demonstrates the ability of intraluminal pulse sampling FLIm to detect severe inflammation, namely, DSS-induced colitis. Current results show that the average fluorescence lifetime was altered in colons with DSS-induced inflammation compared to healthy colons and that 5-ASA treatment returned the lifetime levels closer to baseline. The changes in DSS colons were consistent with the colonocyte metabolic switch from β-oxidation to aerobic glycolysis, characterized by increased intracellular lactate levels and decreased ATP levels and PDH activity. The current findings support further experiments on in vivo models of mild inflammation to assess the sensitivity of intraluminal FLIm as a diagnostic and monitoring tool of early inflammatory disease.

## Data Availability

The data presented in this study are available within the article and on request from the corresponding author.

## Abbreviations

The following abbreviations are used in this manuscript:

FLIm	Fluorescence lifetime imaging
IBD	Inflammatory bowel disease
DSS	Dextran sodium sulfate
5-ASA	5-aminosalicylic acid
NADH	Nicotinamide adenine dinucleotide
FAD	Flavin adenine dinucleotide
